# Microfluidics-Assisted Fabrication of Dual Stopband Photonic Microcapsules and Their Applications for Anticounterfeiting

**DOI:** 10.3390/polym14193954

**Published:** 2022-09-22

**Authors:** Can Zhou, Shoubin Zhang, Taoran Hui, Qiuhong Cui, Yuandu Hu

**Affiliations:** 1 Department of Materials Science and Engineering, School of Physical Sciences and Engineering, Beijing Jiaotong University, Beijing 100044, China; 2 TDK Headway Technologies, Inc., Milpitas, CA 95035, USA; 3 Department of Physics, School of Physical Sciences and Engineering, Beijing Jiaotong University, Beijing 100044, China

**Keywords:** photonic microcapsules, core-shell structure, microfluidics, structural color combination, double photonic stopbands

## Abstract

The assembly of two different kinds of colloidal particle-based photonic structures into an individual micro-object can achieve multifunctionality. In this study, core–shell photonic microcapsules with dual structural colors and photonic stop bands were prepared through a standard microfluidic technique. Photocurable resin suspension of silica nanoparticles and an aqueous suspension of nanogels were used as shell and core parts of microcapsules, respectively. The structural colors of shells and cores can be tuned by adjusting the concentrations of silica nanoparticles and soft nanogels in their corresponding suspensions. The individual microcapsules possess two distinct stop bands when the two suspensions are combined appropriately. Remarkably, the color information of the core part cannot be directly viewed at a macroscopic level (such as visual inspection) but can be detected at a microscopic scale (such as optical microscopy observation). The color information hidden enables the capability for information encryption and has potentially critical applications in anti-counterfeiting, display, and other fields.

## 1. Introduction

A photonic structure is a kind of periodic dielectric structure with periodic sizes on the wavelength scale of visible light [1,2]. The most obvious feature of a photonic structure is the photonic stopband, which prevents the propagation of photons with specific frequencies. In this case, photonic structures exhibit specific and bright colors, generally called ‘structural color’ [3]. The structural color of a photonic structure has received extensive research attention due to its high brightness, high saturation, and color fading resistance [4,5,6]. Generally, there are two approaches to producing photonic structures with structural colors: top-down and bottom-up [7,8]. The top-down route normally requires the use of expensive and sophisticated instruments (i.e., e-Beam or photolithography machine) to generate tiny features on specific substrates, leading to high costs and limited accessibility for most ordinary laboratories. In terms of the bottom-up or self-assembly approach, the photonic structure can be constructed by assembling colloidal particles or amphiphilic block copolymers. This approach is much more affordable and accessible for ordinary laboratories. Comparably, it is a requisite for the block copolymers to have highly precise architectures when used as building blocks for photonic structures [9,10]. This may pose challenges in the perspective of cost-effectiveness and convenience for the preparation process. In light of this, the construction of photonic structures based on colloidal particle-building blocks has been heavily investigated owing to their facile synthetic requirements for colloidal particles. Colloid-based photonic micro-objects with well-defined structures have been intensively studied in recent decades due to their wide applications in pigments, coding–decoding, multiplexing, encryption, and displays [11,12,13,14,15]. Microfluidics is a powerful platform that could produce microdroplets and micro-objects in a highly controllable fashion. The construction of photonic micro-objects by combining microfluidic techniques and assembling colloidal particles has been reported over the past decade [16,17,18]. Colloid-based photonic micro-objects with diverse structures, including microspheres, Janus spheres, microcapsules, and anisotropic microparticles, have since been envisaged and prepared to meet the needs of different applications [19,20,21,22,23,24,25,26,27,28].

In addition to structural regulation (imparting the resulting photonic micro-objects with multi-functionalities), the responsiveness to external stimuli is an essential feature for micro-objects [29,30]. Either the colloidal particle building blocks or the matrix materials used for immobilizing the colloids need to be rendered with stimulus-responsive properties [31,32,33,34,35]. Generally, nondeformable nanoparticles, such as PS, SiO_2_, and magnetic NPs, have been used as building blocks for photonic structures. Those nondeformable NPs either have limited stimuli-responsive properties or complicated synthesis procedures. So, the functionalization of photonic micro-objects heavily relies on the properties of the matrix materials [36,37,38,39,40,41,42]. On the contrary, soft deformable colloidal (such as the classic poly-N-isopropylacrylamide (pNIPAAm)-based nanogels) particle-based photonic suspensions show excellent potential in a variety of fields due to their stimuli-responsive properties, facile multi-functionalities, crystal defect healing properties, etc. [43,44,45,46,47,48]. Both deformable and nondeformable colloidal particle-based photonic micro-objects have been realized through microfluidics. However, most of the resulting photonic micro-objects usually have single optical characteristics, such as single stopbands, limiting further applications [49,50,51,52].

In recent years, photonic micro-objects with dual stopbands and beyond have been envisaged and constructed to realize multiple functionalities of photonic micro-objects [35,53,54]. Among those reports, most dual-stopband photonic micro-objects are achieved through the use of a Janus configuration, constraining their applications in diverse fields. Despite the (few) reports on the construction of dual stopbands and photonic micro-objects with (quasi)-core-shell structures, the colloidal building blocks of these photonic micro-objects all have hard or rigid properties, limiting their multi-functionalities [54]. Construction of those core-shell (or quasi-core/shell)-structured photonic micro-objects with dual stopbands is achieved by methods such as partial etching of SiO_2_ colloidal particles [53], chemical swelling of PS-based colloidal particles [35], or direct encapsulating of photonic dispersion of one hard colloidal particle into another [54]. To the best of our knowledge, the construction of dual-stopband photonic micro-objects by combining photonic dispersion of rigid (or nondeformable) and soft colloidal particles has never been achieved despite considerable merits of the photonic dispersion of soft gel particles.

Herein, we leveraged the merits of both hard and soft colloidal particles and prepared core-shell photonic micro-objects through microfluidics by using an aqueous dispersion of deformable gel particles and photocurable dispersion of hard silica particles as the core-shell parts, respectively. Remarkably, the micro-objects displayed the color from the shell part when observed under visual inspection, while they displayed distinct colors (or two different reflection spectra peaks) from the core-shell parts when observed at a microscopic level. Therefore, when the core–shell photonic microcapsules are used as the building blocks for the preparation of photonic hydrogel films, the visible structural colors of the entire film are only from the colors of the shell of the microcapsules while colors from the shell and core parts can be revealed using microscopic techniques. The micro-objects may have potential applications in the fields of anti-counterfeiting and confidentiality.

## 2. Experimental Section

### 2.1. Material

N-Isopropylacrylamide (NIPAAm, purity ≥ 99%, Sigma-Aldrich, Shanghai, China), *N*,*N*′-Methylene bisacrylamide (BIS, purity ≥ 98%, Sigma-Aldrich), sodium dodecyl sulfate (SDS, purity ≥ 99%, Aldrich, Shanghai, China), Acrylic acid (AAc, purity ≥ 98%, Aladdin, Shanghai, China), Potassium Persulfate (KPS, purity ≥ 99%, Aladdin), ethoxylated trimethylolpropane triacrylate (ETPTA, purity ≥ 99%, Aldrich), poly(vinyl alcohol) (PVA, Mw = 13,000–23,000, 87–89% hydrolyzed, Aldrich), glycerol (purity ≥ 99%, Sinopharm Chemical Reagent Co., Beijing, China), Acrylamide Monomer (AAm, 50% in water, Sinopharm Chemical Reagent Co.), 2-[methoxy(polyethyleneoxy) propyl]trimethoxylsilane (Gelest Inc., Shanghai, China), 2-hydroxy-2-methylpropiophenone (commercial name 1173, purity ≥ 99%, Aldrich), ethanol (AR, purity ≥ 99.7%, Sigma-Aldrich), tetraethyl orthosilicate (purity ≥ 98%, Aladdin), ammonia solution (purity ≥ 28%, NH_3_ in H_2_O, Aladdin), and sodium chloride (AR, purity ≥ 99.5%, Sigma-Aldrich). Pure water was produced from a Mini-Q (Milli-Q Direct, Merck, Shanghai, China) ultrapure water production system.

### 2.2. Synthesis of Nanogel and Preparation of Photonic Nanogel Dispersions with Different Colors

Briefly, 1.06 g of NIPAAm, 0.06 g of BIS, 0.018 g of SDS, and 0.09 g of AAc were dissolved in 70 mL of deionized water in a 250 mL three-necked flask. A three-necked flask fits with a condenser tube, nitrogen was introduced, and then heated to 70 °C in an oil bath. KPS (0.03 g) dissolved in 10 mL of water was added dropwise to the mixture solution to initiate polymerization. Under continuous stirring and nitrogen bubbling, the reaction was carried out for four hours. The synthesized nanogel dispersion product was cooled to room temperature after the reaction.

The concentrated nanogel suspensions with different colors were obtained by evaporating about 10 g of nanogel suspension at 70 °C. The colors of the suspensions depended on the concentrations of nanogels in the dispersion. Three typical structural colors, blue, green, and red, originated, containing 4.2%, 3.2%, and 2.2% of nanogels, respectively. By controlling the evaporation time, the concentrations of nanogel in suspension could be adjusted, yielding different colors of nanogel suspensions [43].

### 2.3. Preparation of Resin Suspension of Silica Nanoparticles

A total of 85 mL of ethanol, 4 mL of ammonia water, and 8 mL of deionized water were put into a 250 mL conical flask, magnetically stirring at a constant temperature of 30 °C. A total of 3 mL of TEOS was added after the temperature was stabilized. The mixture solution was stirred at 700 rpm., and 15 min later, the stirring speed was adjusted to 500 rpm when the solution became milky white. The hydrolysis reaction was maintained at a constant temperature of 30 °C for six hours. After the reaction was complete, the product solution was centrifuged at 8000 rpm for 6 min. The supernatant solution was removed, leaving behind the centrifuged nanoparticles. Ethanol was added to the centrifuge tubes to redisperse the centrifuged nanoparticles with sonication. This centrifugation-redispersion process was repeated three times to purify the silica nanoparticles. Eventually, silica nanoparticles with relatively narrow size distributions could be obtained. We reduced the reaction time and adjusted the amount of ammonia water to synthesize the silica nanoparticles with different diameters.

The purified silica nanoparticle in ethanol was mixed with different amounts of ETPTA and a tiny amount of 2-hydroxy-2-methyl propiophenone (0.5% to the total weight of the silica NPs and ETPTA resin) to obtain a mixture solution [41]. The mixture solution was heated in an oven at 70 °C for 12 h to completely evaporate the ethanol, yielding a resin suspension of silica nanoparticles with different colors. The blue, green, and red silica suspensions were composed of silica particles at particle concentrations of φ = 0.33, φ = 0.25, and φ = 0.17, respectively. The chemical structure of ETPTA is shown in Figure S1.

### 2.4. Construction of Microfluidic Device

**Figure 1:**
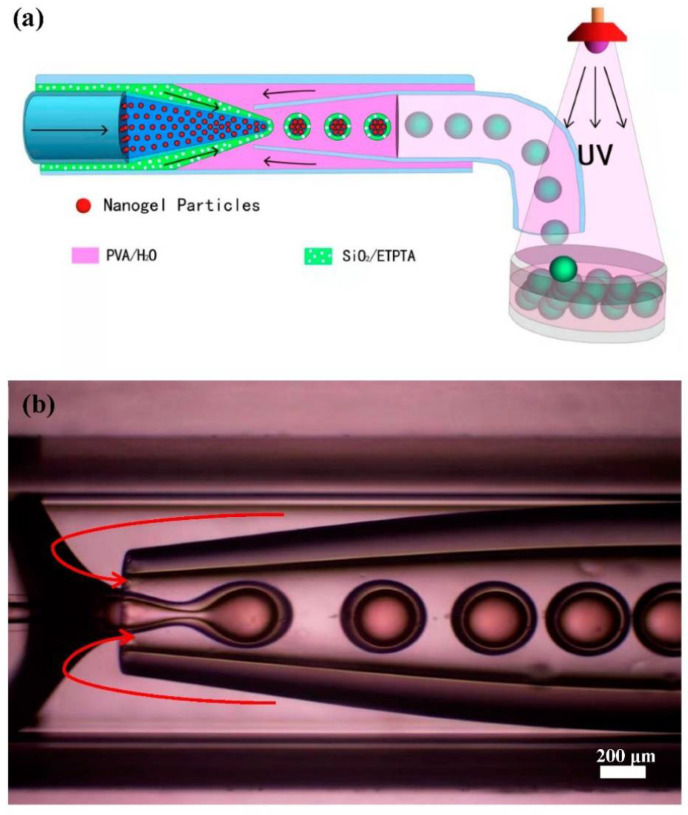
(**a**) Schematic illustration of the microfluidic fabrication process of the core-shell photonic microcapsules. (**b**) Optical microscopy image of the core-shell photonic microdroplet fabrication process inside a glass capillary-based microfluidic device.

The microfluidic device was constructed by assembling silane-treated glass capillaries of different geometries and orifices. Epoxy resin was used to seal the glass capillaries and injection needles. As shown in Figure 1, the round glass capillary (outer diameter (OD): 1 mm, world precision instruments) was heated to obtain two blunt capillaries, and then their blunt mouth sizes were processed to prepare two round capillaries with a small orifice of about 100 µm and a relatively larger orifice of about 300 µm, respectively. The capillary with the larger orifice was treated hydrophilically using 2-[methoxy(polyethyleneoxy) propyl]trimethoxylsilane. Finally, the two processed round capillaries were coaxially inserted into the square glass tube (inner dimension: 1.05 mm × 1.05 mm, AIT glass), and the blunt mouths of the two capillaries were kept at a certain separation of ~60 µm. They were assembled with the syringe needle and sealed with epoxy resin.

### 2.5. Preparation of Thin Films Containing Photonic Microcapsules

Briefly, 1 mL of AAm solution (weight percentage of 50%), BIS (2 wt% compared to the pure weight of AAm), and photoinitiator 2-hydroxy-2-methylpropiophenone (0.5 wt% compared to the pure weight of AAm) were slowly added to 2 mL of deionized water. The aqueous solution was added to a glass petri dish. Subsequently, the desired amounts of photonic microcapsules were dispersed in the aqueous solution to obtain a monolayer assembled structure by gentle handshaking. The solution mixture was then polymerized under UV light irradiation (Jiapeng ZF-5, 16 W, Shanghai, China) for 5 min.

### 2.6. Optical Microscopy

An inverted optical microscope (LWD300-38LT, Beijing CeWei Optoelectronics, Beijing, China) was used to monitor the formation of emulsion droplets in the microfluidic device under a bright field mode (note: the light should not be too strong given that strong light could trigger pre-curing of the resin at the tip of the injection tube, resulting in the blocking of the microfluidic device). The images were captured by a high-speed CCD (20 MP, USB 3.0) that was attached to the microscope. The structural colors of the core–shell photonic microcapsules were observed under the reflection mode using an upright optical microscope (Olympus BX53M, Olympus Corporation, Beijing, China). Images were captured by a digital camera attached to the microscope. Finally, we used stream software for data collection and analysis.

### 2.7. Scanning Electron Microscope (SEM) and Transmission Electron Microscope (TEM)

The silica nanoparticles were placed on a silicon wafer for sample preparation, and their surface morphologies were imaged by SEM (Sirion 200, FEI). The prepared core-shell photonic microcapsules were cut in half with a blade to observe the multi-directional internal morphology. The non-concentrated nanogel suspension was purified by dialysis for 14 days and diluted 3-fold. A drop of diluted nanogel suspension was spread on the TEM grid and freeze-dried. The sample was stained with 1 wt% of phosphotungstic acid aqueous solution and then imaged by TEM (Tecnai G2 20, FEI).

### 2.8. Fiber Optical Spectra

The reflection spectrum of the core-shell photonic microcapsules was measured by a fiber optic spectrometer (USB Flame-S, Ocean Insight, Inc., Shanghai, China) equipped with an optical microscope (Olympus BX53M).

## 3. Results and Discussion

### 3.1. Preparation of Core-Shell Photonic Microcapsules Using Soft and Hard Colloidal Nanoparticles as Building Blocks

Monodispersed pNIPAm-co-AAc nanogels and silica particles with different particle sizes were first synthesized using the conventional precipitation polymerization method and sol–gel method, respectively. SEM and TEM characterizations of silica and nanogel particles are shown in Figure S2. It can be seen that the two partial colloidal particles (as structural units) have a uniform size and regular distribution. The synthesized and purified silica particles in ethanol solution were further dispersed in a photocurable ethoxylated trimethyl propane triacrylate (ETPTA) resin. Evaporation-induced colloidal self-assembly can result in the formation of resin dispersion of silica nanoparticles with different structural colors. The suspension was used as the middle phase in microfluidic experiments. The synthesized gel particle suspension was also subjected to solution evaporation, and the evaporation time was controlled to induce the formation of photonic suspension, which was subsequently used as the inner phase. A standard microfluidic experiment was illustrated in Figure 1a, where 10 wt.% of PVA aqueous solution was used as the outer phase. The three liquids were injected into a glass capillary-based microfluidic device.

Core-shell emulsion droplets were formed by the shear action of oil in another aqueous phase, specifically the outer aqueous phase [16,43,45]. The hydrogel suspension was delivered through the left inner capillary. The silica-in-ETPTA suspension was fed as an intermediate phase through the annulus region between the inner and outer capillaries on the left, and the aqueous phase flowed through the annulus region between the inner and outer capillaries on the right. When the aqueous phase flowed to the tip of the capillary, it broke the stream of the silica suspension and hydrogel suspension. The shearing effect induced emulsification to form oil-in-water emulsified droplets so that the silica-in-ETPTA suspension encapsulated the soft gel nanoparticle suspension to form photonic microcapsules with a core–shell structure. By adjusting the flow rates of the three phases, the core-shell drops with uniform sizes could be produced. The droplets were collected in a Petri dish, and the shell part was cured under an ultraviolet lamp to form stable photonic micro-objects. It can be seen from Figure 1b that the shear force generated by the flow of the water phase on the right could cause the silica suspension to wrap the hydrogel particle suspension and form core-shell droplet microcapsules.

**Figure 2:**
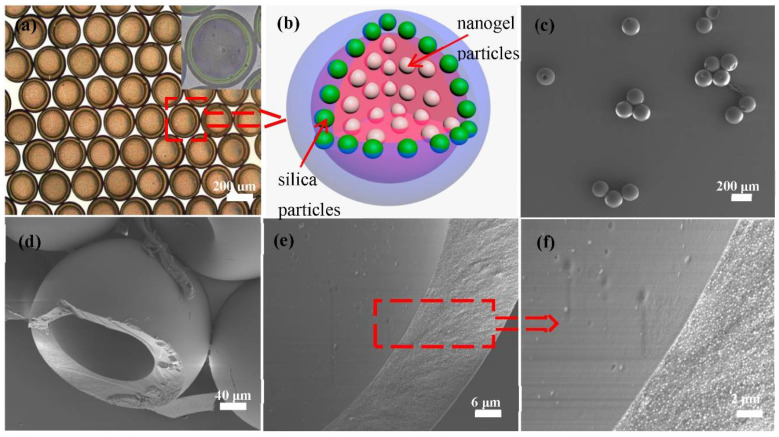
(**a**) Optical microscope image of the core-shell microcapsules. (**b**) Schematic illustration of the structure of the photonic microcapsule. (**c**) SEM image of several photonic microcapsules. (**d**–**f**) SEM images of the internal structure and cross-section of a microcapsule. (**f**) Magnified image of the inner surface and cross-section of the microcapsule in (**e**).

Core-shell photonic microcapsules with different sizes and shell thicknesses can be produced from the microfluidic device by adjusting simple parameters, such as the injection flow rate of the inner, middle, and outer phases (Figure S3). Typical core-shell photonic microcapsules with high monodispersity (as shown in Figure 2a,b) were prepared. Figure 2b illustrates the structures of the resulting microcapsules.

As shown in Figure 2c, it can see that the photonic microcapsules under SEM scan maintained good integrity due to the photocuring-induced formation of the solid shell. The silica particles on the surface of the microcapsules were regularly embedded in the ETPTA resin matrix (Figure S4). The cross-section SEM images of the microsphere (after cutting the photonic microcapsules) are shown in Figure 2d–f. Clearly, the core-shell structure of the microcapsules can be seen from the images, where the inner surface of the microcapsule is also relatively smooth. However, due to the drying process during the standard sample preparation process, the inner soft hydrogel particles were precipitated onto the inner surface and may have become lost during the sample transferring process or vacuuming process prior to SEM characterization. As a result, the nanogels were not observed in our characterizations.

### 3.2. Photonic Microcapsules with Different Structural Color Combinations from the Core and Shell Parts

Hard silica nanoparticles can be dispersed in the ETPTA resin to form dispersions with different photonic properties, the frequencies of which reflect light, depending on the sizes and concentrations of the nanoparticles [41]. Likewise, soft gel suspension structural colors are determined by the volume fractions of nanogels in the suspension and can be easily tuned by changing the concentration of nanogels [43]. This can be explained by the Bragg law: nλ = 2d sinθ, where n is the effective refractive index, λ is the wavelength, d is the distance between adjacent crystal planes, and θ is the viewing angle. Clearly, d decreases as the concentration of soft gel nanoparticles in the suspension increases, resulting in a blue shift of the reflection peak, and vice versa. This is also the principle of the structural color situation of silica nanoparticles dispersed in the resin suspension. On those bases, gel particle suspensions with three structural colors (red, green, and blue) can be prepared by controlling the evaporation time (Figure S5a). Similarly, when different volume fractions of silica particles are dispersed in a certain amount of ETPTA resin, the photonic suspension of silica NPs with red, green, and blue colors can be obtained. Their structural colors and reflection spectra images are self-explanatory (Figure S5b–e).

**Figure 3:**
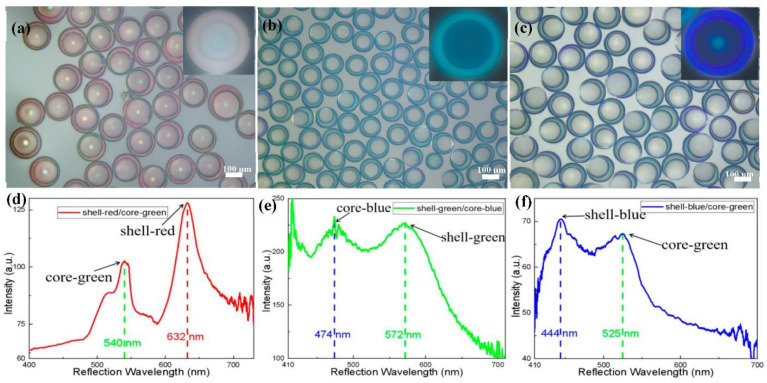
(**a**–**c**) Optical microscope images of photonic microcapsules under reflection mode with three structural color combinations. (**a**): shell-red/core-green, (**b**): shell-green/core-blue, (**c**): shell-blue/core-green. (**d**–**f**) Reflectance spectra of the corresponding photonic microcapsules with different structural color combinations.

Based on the above conditions, to realize photonic microcapsules with double stopbands, ETPTA resin suspensions of silica nanoparticles and aqueous suspensions of soft nanogel particles were used as the outer shell and inner core, respectively. The presence of SDS molecules in the core part could minimize the interfacial tension between the nanogel dispersion and resin suspension. As a result, stable core-shell emulsion droplets can be produced from the microfluidic emulsification step, eventually yielding core-shell microcapsules upon UV irradiation. The corresponding core-shell photonic microcapsules were prepared by various combinations with different structural colors of two different suspensions, as shown in the optical microscope image in Figure 3. Three different typical color combinations were demonstrated in our study: (a) the red shell and green core, (b) the green shell and blue core, and (c) the blue shell and green core. The combinations of the resulting microcapsules are shown in Figure 3a–c, respectively. The distinct shell structure can be seen in the reflection mode of the optical microscope. The structural colors of the shell and inner core are very bright and different (as shown in Figure S6, where the microcapsules were dispersed in aqueous solutions of NaCl). It can be seen from the reflection spectra of the three structural color combinations in Figure 3d–f that there were two characteristic peaks in these spectra, with maximum reflection peaks at 540 and 632 nm for microcapsule a, 474 and 572 nm for microcapsule b, and 444 and 525 nm for microcapsule c. The wavelength peak values well corresponded to the structural colors of the shell and inner core of the photonic microcapsules. This phenomenon is more favorable to proving that the optical properties of the double stopbands of core-shell photonic microcapsules can be realized through the combination of soft and hard nanoparticles. Its responsiveness and functionalization will be more comprehensive.

In addition, it is also possible to produce core-shell structures using the two different suspensions with similar structural colors (as shown in Figure S7). However, the two peaks in the reflection spectrum of this specific microcapsule are relatively close, resulting in a broad reflection spectrum peak (as shown in Figure S7d,e). The silica shell structures of photonic microcapsules after photopolymerization are hard and stable. In addition, the structural colors of the photonic microcapsules dispersed in water for nine months are still evident under an optical microscope. Moreover, their shapes and monodispersity do not change (as shown in Figure S8). The purpose of our research is that when the hard silica shell adopts one structural color, the soft gel core part can have a different (or the same) structural color to realize the combination of soft and hard nanoparticles with double-stopband core-shell photonic microcapsules. The core-shell photonic microspheres prepared in this fashion not only possess the nondeformable and optical properties of hard silica particles but also have the merits of soft hydrogel particles, which will be in our upcoming detailed study. These structural colors can also be further verified by fiber optic spectrometer testing.

### 3.3. Performances of Hydrogel Films Containing Photonic Microcapsules

**Figure 4:**
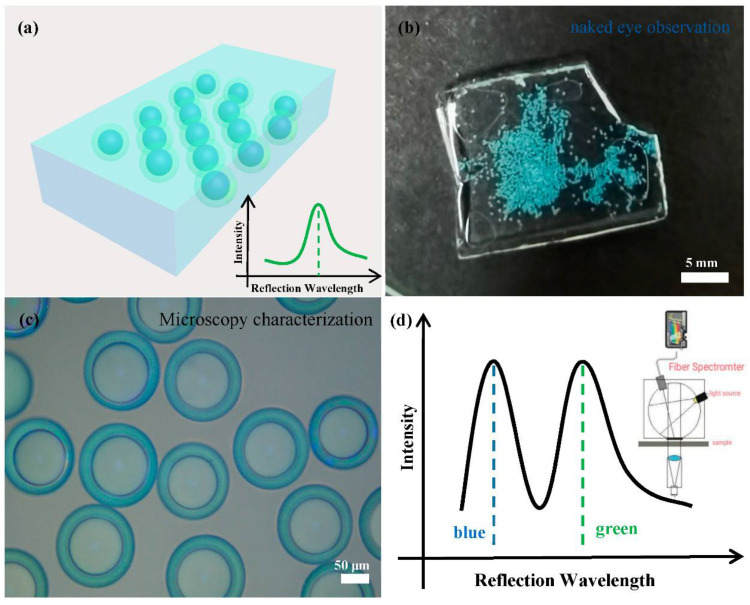
(**a**) Schematic diagram of the film observed with the naked eye and the reflection spectrum diagram. (**b**) Photographs of core–shell photonic microcapsules dispersed in a poly(acrylamide) hydrogel film. The structural color combinations of the photonic microcapsules are shell-green/core-blue. (**c**) The optical microscope image of the thin film in reflection mode. (**d**) Schematic diagram of the reflection spectrum of the thin film measured by the fiber optic spectrometer.

The microfluidic technique allows us to produce such core-shell photonic microcapsules in large quantities in a controlled manner, facilitating us to utilize the microcapsules for secondary assembly. Specifically, the as-prepared photonic microcapsules can be immobilized in a hydrogel film for practical applications. In particular, hydrogel film embedded with a green shell and blue core microcapsules was used for a demonstration, as shown in Figure 4. It can be seen that the color of the hydrogel film is clearly visible to the naked eye, which is the structural color of the green shell microcapsules. The photonic microspheres were tightly arranged and in a stable state. Our naked eye cannot directly visualize the color from the inner core part. However, the color from the inner core part became apparent when the hydrogel film was placed under an optical microscope or characterized by a fiber spectrometer. Two distinct colors or distinct reflection spectrum peaks from the microcapsules can be detected. This phenomenon suggests that the microcapsules may have great potential application in anti-counterfeiting and other photonic crystals.

## 4. Conclusions

In this study, we prepared monodispersed core-shell photonic microcapsules with double stopbands by combining soft gel particles and rigid silica nanoparticles through a microfluidic technique. The shell of the microcapsules was composed of photocurable resin dispersion of silica nanoparticles, while the inner core part was made of an aqueous dispersion of soft gel nanoparticles. Both dispersions have photonic properties. The core-shell structure could combine the advantages of rigid nanoparticles and soft deformable gel particles. Then dual-stopband microcapsules, a combination of distinct structural colors from the shell and core parts, could be achieved. The two combined parts of the core–shell microcapsules, respectively, exhibited excellent structural colors and optical properties at the microscale. The research results show that encapsulating soft gel particles in photocurable resin suspension (of hard nanoparticle) shell microspheres to form a core-shell structure could realize optical properties, such as double stopbands and multi-functionalization. In addition, the microcapsules can be further served as building blocks for secondary assembly and form photonic hydrogel films when being embedded in a transparent hydrogel matrix. Interestingly, the color information only originating from the shell parts of the microcapsules could be viewed using our naked eye, while the color information from the shell and core parts could be detected under the microscopic characterizations. This may allow us to leverage this feature to construct information encryption devices. Moreover, microcapsules may have important potential applications in the fields of anti-counterfeiting technology and confidentiality.

## Data Availability

The data presented in this study are available upon request from the corresponding author.

## Supplementary Materials

The following supporting information can be downloaded at: https://www.mdpi.com/article/10.3390/polym14193954/s1, Figure S1: chemical structural of ETPTA (ethoxylated trimethylolpropane triacrylate); Figure S2: (a–c) SEM images of silica particles of different sizes; (d) SEM image of Nanogel particles; (e) TEM images of Nanogel particles. (f) Schematic diagram of the size distribution of silica nanoparticles; Figure S3: (a) Image of thin-shelled PC microspheres fabricated by microfluidic device; (b–d) Images of core-shell PC microspheres of different sizes and shell thicknesses under optical microscopy; Figure S4: SEM image of core-shell PC microspheres. (a,b) External surface morphology of microspheres; (c–e) Enlarged image of the shell and inner surface of the microspheres; Figure S5: (a) Soft nanogel suspension—three structure colors: blue, green, red. They contain 4.2%, 3.2%, and 2.2% of nanogels. (b) Suspension of silica particles dispersed in ETPTA resin—three structural colors: blue, green and red. These silica suspensions were composed of silica particles at a particle concentration of φ = 0.33, φ = 0.25, and φ = 0.17. (c–e) Reflectance spectra of suspensions of three structural colors; Figure S6: Images of core-shell PC microspheres with different structural colors dispersed in Sodium chloride aqueous solution; Figure S7: (a–c): core-shell PC microspheres with the same core-shell structure color. (d–e) Corresponding reflectance spectrum images; Figure S8: (a) Microscopic image of freshly prepared photonic microcapsules in reflection mode. (b) Microscope image of photonic microcapsules in reflection mode after being placed in an aqueous solution for nine months. Both are the shell-red/core-green.
