# Supplementary material for: Microfluidics-Assisted Fabrication of Dual Stopband Photonic Microcapsules and Their Applications for Anticounterfeiting
Source: Polymers (Basel). 2022 Sep 22;14(19):3954. doi: 10.3390/polym14193954 (PMC9572925; doi:10.3390/polym14193954)
Supplement: Supplementary file 1 [file polymers-14-03954-s001.zip › polymers-1917979-supplementary.pdf]

Supporting information

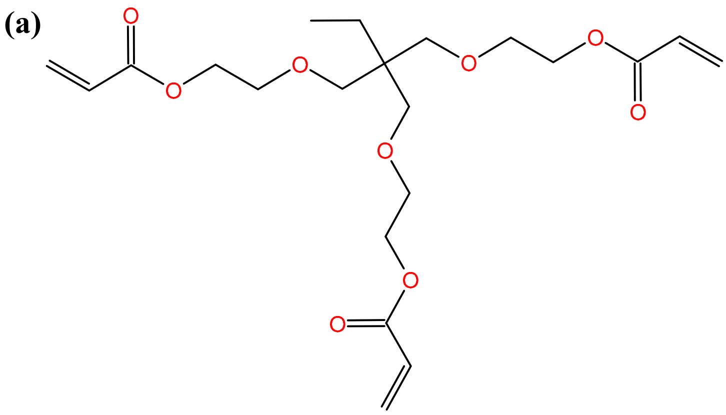

Figure S1: chemical structural of ETPTA(ethoxylated trimethylolpropane triacrylate)

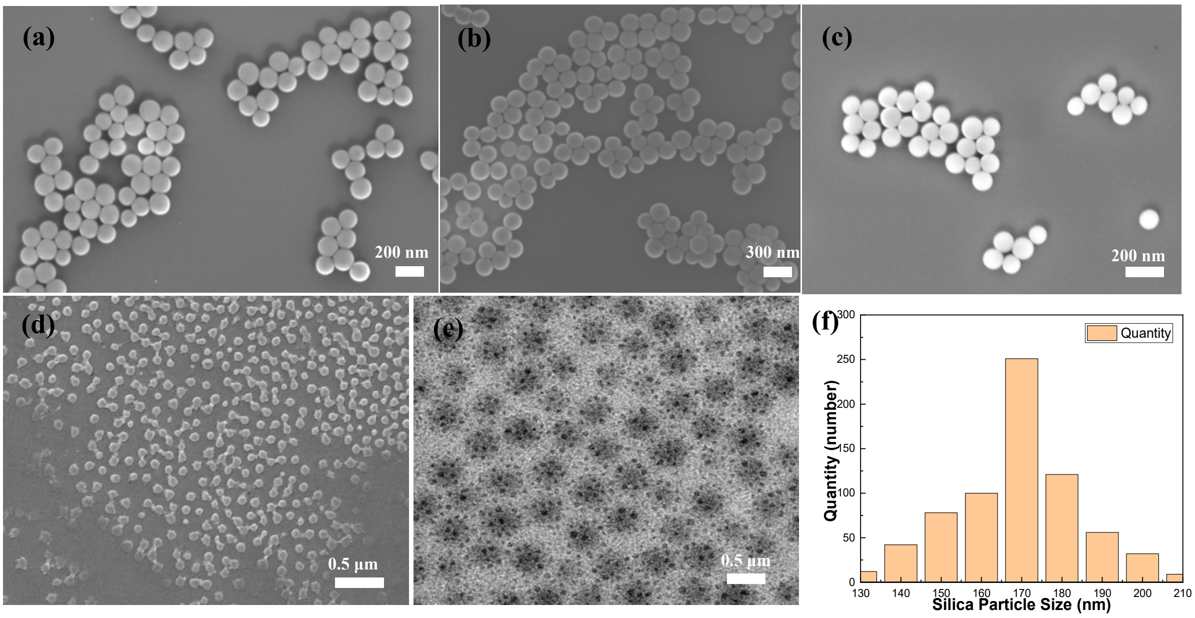

Figure S2: (a-c) SEM images of silica particles of different sizes; (d) SEM image of Nanogel particles; (e) TEM images of Nanogel particles. (f) Schematic diagram of the size distribution of silica nanoparticles.

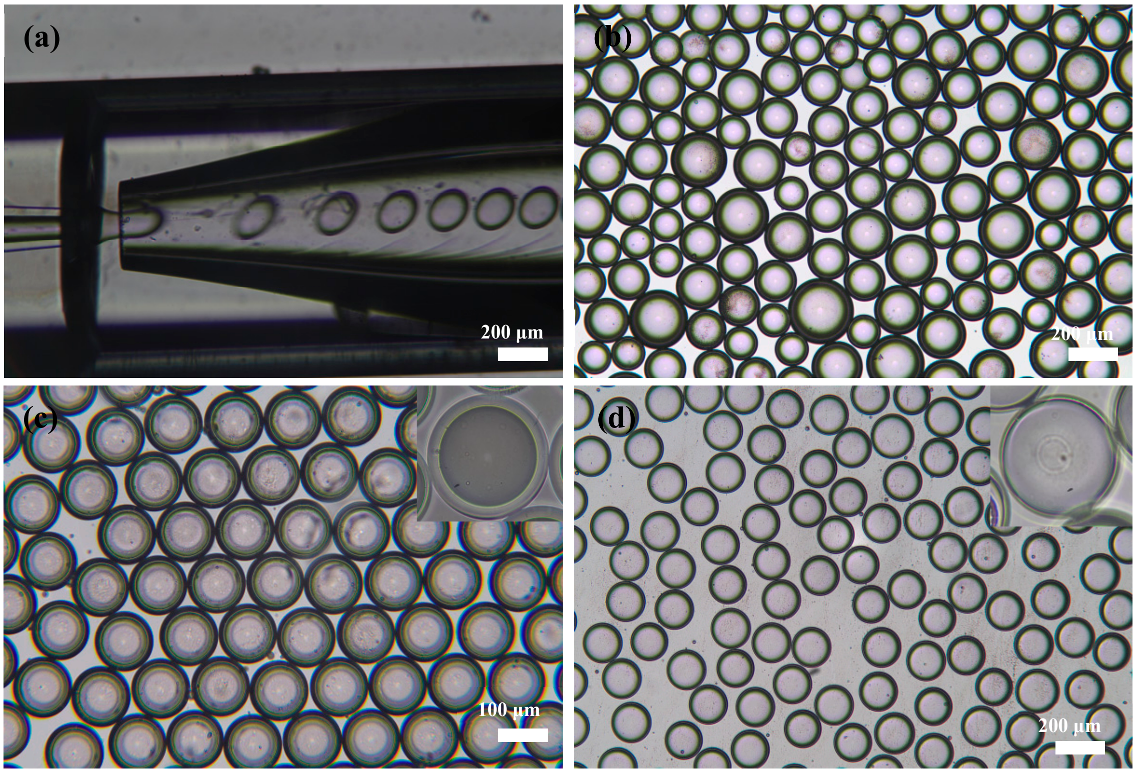

Figure S3: (a) Image of thin-shelled PC microspheres fabricated by microfluidic device; (b-d) Images of core-shell PC microspheres of different sizes and shell thicknesses under optical microscopy.

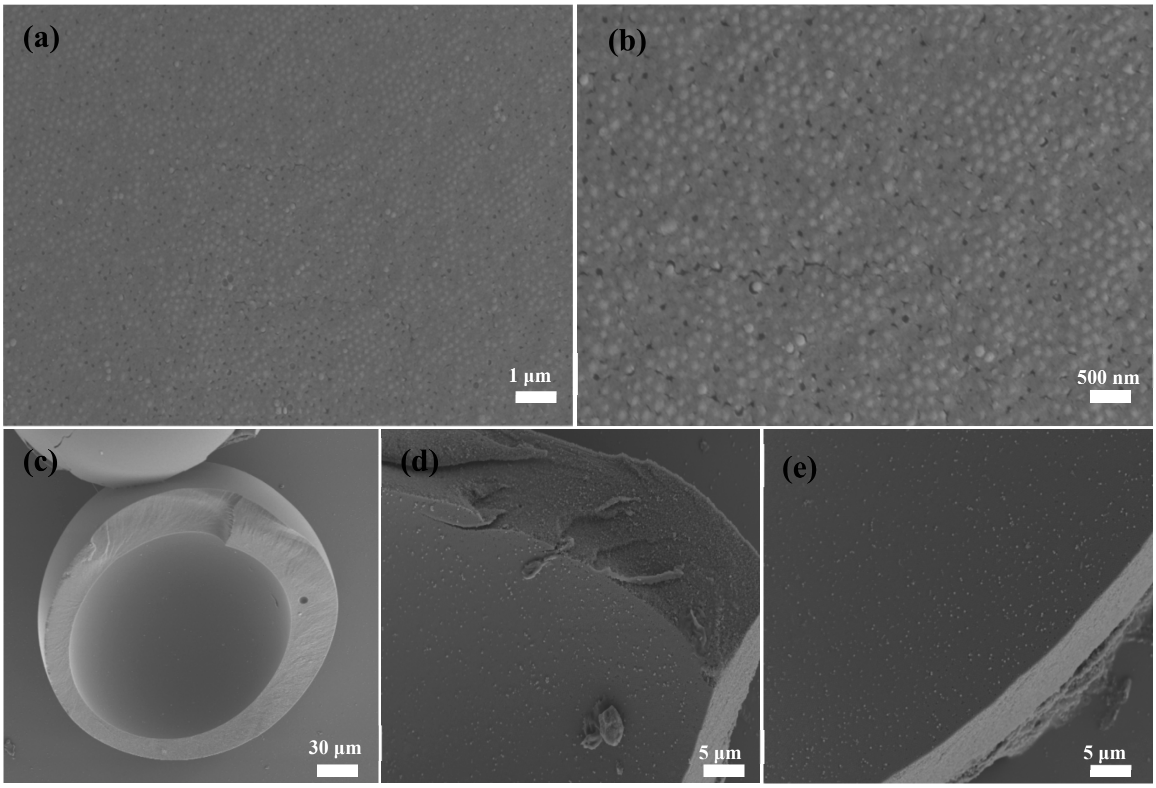

Figure S4: SEM image of core-shell PC microspheres. (a),(b) External surface

morphology of microspheres; (c-e) Enlarged image of the shell and inner surface of the microspheres.

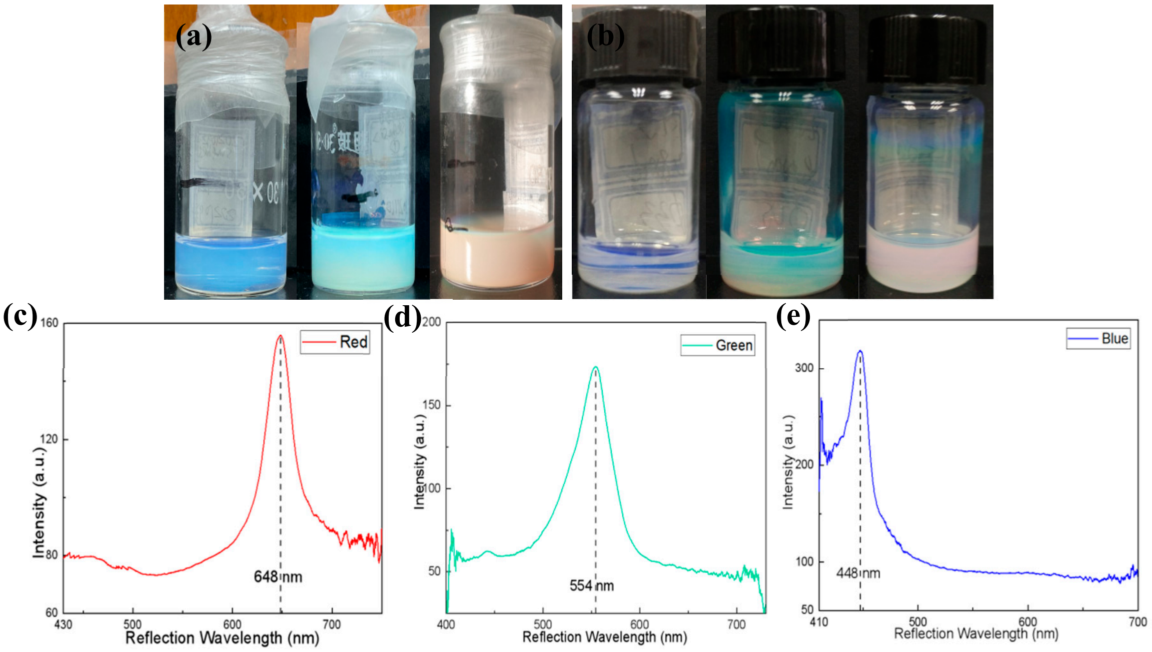

Figure S5: (a)Soft nanogel suspension — three structure colors: blue, green, red. They contain 4.2%, 3.2%, and 2.2% of nanogels. (b)Suspension of silica particles dispersed in ETPTA resin — three structural colors: blue, green and red. These silica suspensions were composed of silica particles at a particle concentration of $\Phi = 0.33$, $\Phi = 0.25$, and $\Phi = 0.17$. (c-e) Reflectance spectra of suspensions of three structural colors.

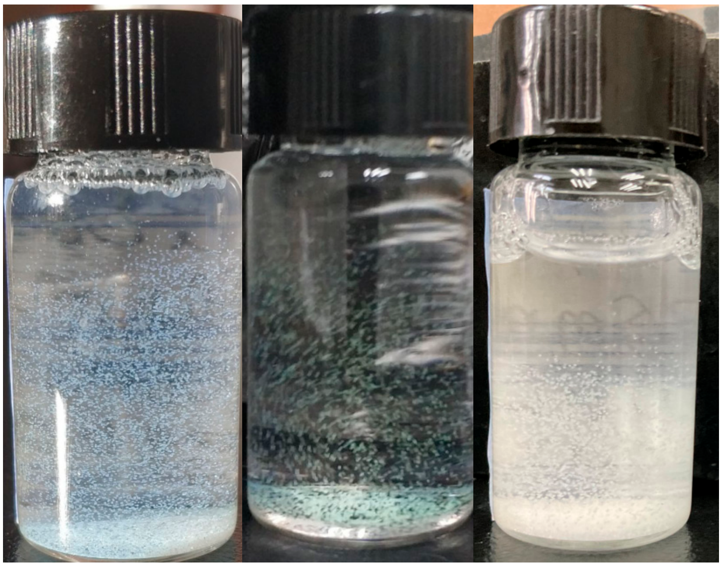

Figure S6: Images of core-shell PC microspheres with different structural colors dispersed in Sodium chloride aqueous solution.

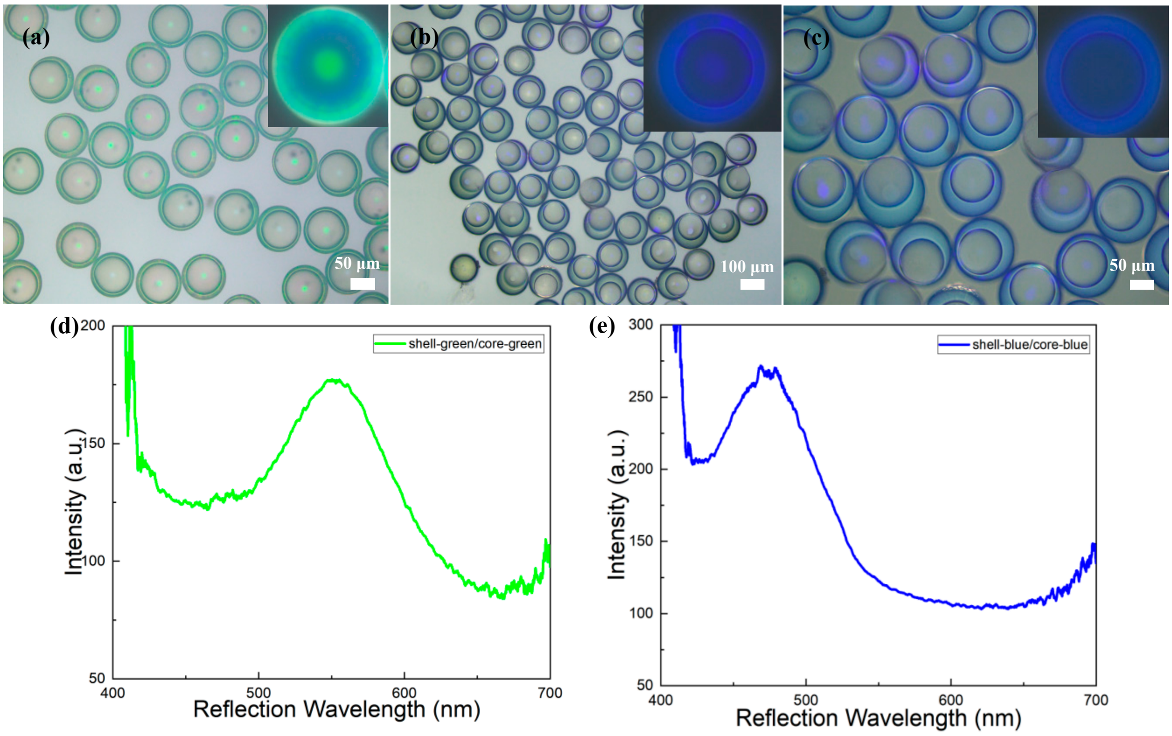

Figure S7: (a-c): core-shell PC microspheres with the same core-shell structure color. (d-e) Corresponding reflectance spectrum images.

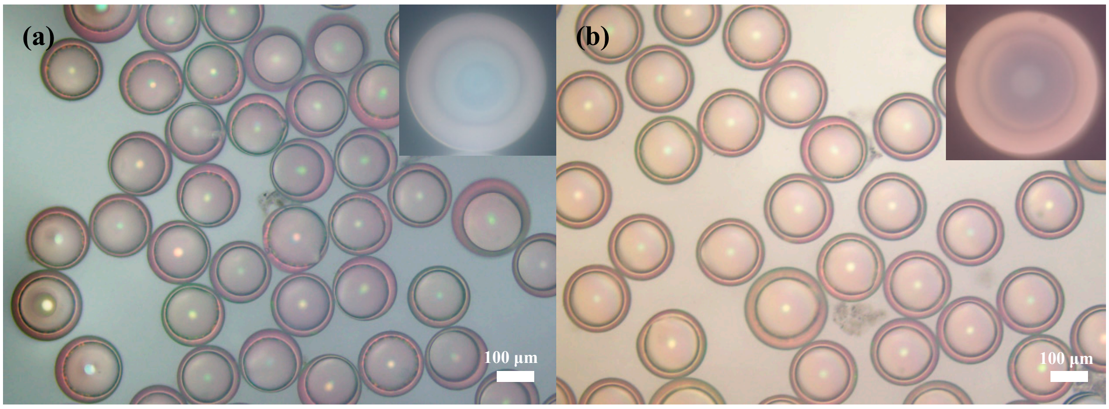

Figure S8：(a) Microscopic image of freshly prepared photonic microcapsules in reflection mode. (b) Microscope image of photonic microcapsules in reflection mode after being placed in an aqueous solution for nine months. Both are the shell-red/core-green.
